# Unravelling the alcohol harm paradox: a population-based study of social gradients across very heavy drinking thresholds

**DOI:** 10.1186/s12889-016-3265-9

**Published:** 2016-07-19

**Authors:** Dan Lewer, Petra Meier, Emma Beard, Sadie Boniface, Eileen Kaner

**Affiliations:** Imperial College Healthcare NHS Trust, Charing Cross Hospital, London, W6 8RF UK; ScHARR, University of Sheffield, Regent Court, 30 Regent Street, Sheffield, S1 4DA UK; Department of Epidemiology & Public Health and Department of Clinical, Educational and Health Psychology, University College London, 1-19 Torrington Place, London, WC1E 6BT UK; National Addications Centre, Institute of Psychiatry, Psychology and Neuroscience, King’s College London, 4 Windsor Walk, London, SE5 8BB UK; Institute of Health and Society, Newcastle University, Baddiley-Clark Building, Richardson Road, Newcastle upon Tyne, NE2 4AX UK

## Abstract

**Background:**

There is consistent evidence that individuals in higher socioeconomic status groups are more likely to report exceeding recommended drinking limits, but those in lower socioeconomic status groups experience more alcohol-related harm. This has been called the ‘alcohol harm paradox’. Such studies typically use standard cut-offs to define heavy drinking, which are exceeded by a large proportion of adults. Our study pools data from six years (2008–2013) of the population-based Health Survey for England to test whether the socioeconomic distribution of more extreme levels of drinking could help explain the paradox.

**Methods:**

The study included 51,498 adults from a representative sample of the adult population of England for a cross-sectional analysis of associations between socioeconomic status and self-reported drinking. Heavy weekly drinking was measured at four thresholds, ranging from 112 g+/168 g + (alcohol for women/men, or 14/21 UK standard units) to 680 g+/880 g + (or 85/110 UK standard units) per week. Heavy episodic drinking was also measured at four thresholds, from 48 g+/64 g + (or 6/8 UK standard units) to 192 g+/256 g + (or 24/32 UK standard units) in one day. Socioeconomic status indicators were equivalised household income, education, occupation and neighbourhood deprivation.

**Results:**

Lower socioeconomic status was associated with lower likelihoods of exceeding recommended limits for weekly and episodic drinking, and higher likelihoods of exceeding more extreme thresholds. For example, participants in routine or manual occupations had 0.65 (95 % CI 0.57–0.74) times the odds of exceeding the recommended weekly limit compared to those in ‘higher managerial’ occupations, and 2.15 (95 % CI 1.06–4.36) times the odds of exceeding the highest threshold. Similarly, participants in the lowest income quintile had 0.60 (95 % CI 0.52–0.69) times the odds of exceeding the recommended weekly limit when compared to the highest quintile, and 2.30 (95 % CI 1.28–4.13) times the odds of exceeding the highest threshold.

**Conclusions:**

Low socioeconomic status groups are more likely to drink at extreme levels, which may partially explain the alcohol harm paradox. Policies that address alcohol-related health inequalities need to consider extreme drinking levels in some sub-groups that may be associated with multiple markers of deprivation. This will require a more disaggregated understanding of drinking practices.

## Background

Excessive alcohol use contributes to over 60 disease conditions and is responsible for 5 % of global disability-adjusted life-years lost [1]. Treatment of alcohol-related health problems has been estimated to account for 9 to 23 % of healthcare costs in a selection of high-income countries [2]. As well as creating a large burden on health and healthcare services, alcohol is a strong driver of health inequality.

Many studies in high-income countries have shown that alcohol-related morbidity and mortality is more common in people of low socioeconomic status (SES) [3–7]. However, cross-sectional surveys regularly show that lower SES groups report drinking the same or less on average than higher SES groups, and are more likely to report abstaining altogether [8–11]. This has been called the ‘alcohol harm paradox’. It has been observed in many countries including the UK, [12] Australia, [13, 14] the Netherlands [15] and Finland [16]. An international meta-analysis showed that people with lower levels of education have higher rates of alcohol-related disease that are not explained by consumption patterns [3].

Various theories have been proposed to explain this paradox, [12] including recall and selection bias in self-report surveys of drinking, greater vulnerability to harm of low SES groups due to co-morbidities or clustering of risk factors with multiplicative effects [17] and differential access to health services. One theory that has not been well tested regards the distribution of drinking levels within SES groups and particularly the distribution of more extreme drinking. Aggregate data may mask the fact that low SES groups may include both more abstainers and light drinkers as well as more extreme, heavy drinkers.

Most UK studies define heavy episodic, high intensity or ‘binge’ drinking as 48 g or more of pure alcohol in one day for women and 64 g or more for men (6 UK units for women and 8 for men). Prior to January 2016, the UK government’s recommended ‘low risk’ limit for weekly drinking was 112 g for women and 168 g for men (14 UK standard drink units for women and 21 for men) [18]. However, 24 % of men and 18 % of women report exceeding the low risk limit for weekly drinking [8]. Little is known about the range of drinking practices within this large population subgroup. The paucity of research into high-intensity drinking has also been observed in the US, leading to calls for a deeper understanding of the characteristics of people drinking far beyond the levels usually studied [19].

This study examined social gradients in extreme alcohol consumption. We hypothesised that (a) higher SES groups would be more likely to exceed standard thresholds, as observed in other studies, and (b) lower SES groups would be more likely to exceed more extreme levels of drinking, as this may help explain the higher rates of harm in these groups. The study is set in England, where the alcohol harm paradox has been clearly observed and there is sufficient survey data to study rarely reported extreme levels of drinking.

## Methods

### Data source

The data source was the Health Survey for England, an annual cross-sectional survey representative of adults living in private households in England. The survey has collected information on adult alcohol consumption since 1991. The full methodology is described elsewhere [20]. Only participants aged 18 or over were included. Six years of data (2008–2013) were combined to increase precision of analyses. Response rates across these survey years ranged from 64 to 68 %.

A beverage-specific method was used to measure alcohol consumption. Participants were asked about the size and number of drinks for seven types of alcohol (beer/cider, strong beer/cider, spirits/liqeurs, fortified wine, wine, alcopops and ‘other’). The questions were repeated for the heaviest drinking day in the past week (beverage-specific recent recall) and a typical drinking day in the past 12 months (using a beverage-specific quantity-frequency measure, which, allowed calculation of typical weekly drinking).

### Outcome variables

Two types of drinking behaviour were examined: heavy weekly drinking (the total drinking across a week) and heavy episodic drinking (the maximum in any one day in the past week). For both types, four dichotomous outcome variables were generated, showing whether or not the individual’s drinking exceeded successive thresholds (see Table 1, which also shows the measures in terms of standard UK units).

**Table 1:** drinking thresholds (women/men), in grams of pure alcohol and UK units. 1 UK unit = 10 ml or 8 g of pure alcohol

	Heavy episodic		Heavy weekly	
Threshold	Grams	UK units	Grams	UK units
1	48+/64+	6+/8+	112+/168+	14+/21+
2	96+/128+	12+/16+	280+/400+	35+/50+
3	144+/192+	18+/24+	480+/640+	60+/80+
4	192+/256+	24+/32+	680+/880+	85+/110+

For weekly drinking, the four thresholds were (1) exceeding the UK government’s recommended (prior to January 2016) weekly ‘low risk’ limit: 112 g or more for women and 168 g or more for men, [18] (2) the UK government’s definition of ‘higher risk’ weekly drinking: 280 g or more for women and 400 g or more for men, [21] (3) 480 g or more for women and 640 g or more for men, (4) 680 g or more for women and 880 g or more for men. Only the most recent three survey years (2011–2013) were combined for weekly drinking, because questions allowing estimation of a full week’s consumption were introduced in 2011.

For heavy episodic drinking, the four thresholds were (1) the UK government’s ‘binge’ definition: 48 g or more of pure alcohol on the heaviest day in the past week for women and 64 g or more men, [22] (2) 96 g or more for women and 128 g or more for men, (3) 144 g or more for women and 192 g or more for men, (4) 192 g or more for women and 256 g or more for men.

The patterns of alcohol consumption in terms of sex, age and ethnicity were similar to those observed in other surveys [11, 23].

### Socioeconomic status indicators

SES indicators from the Health Survey for England were: (1) equivalised income quintile. ‘Equivalised income’ is the total household income adjusted according to household members. Households with children would have their income adjusted down, for example. (2) Highest educational qualification, summarised as degree (14+ years of education), A-level (13 years), GCSE (11 years), other/foreign and none. (3) Occupation, using the National Statistics Socioeconomic Classification, [24] based on the respondent’s ‘household reference person’ (the person who owns or rents the house or who has the highest income). (4) Neighbourhood deprivation quintile, based on the Index of Multiple Deprivation [25]. This is an area-based index derived from levels of income, employment, health, disability, education, skills and training in small local areas with roughly 1500 residents. The 2007 version of the index was used for survey years 2008–2010. The 2010 version was used for survey years 2011–2013.

### Analyses

Data were complete apart from alcohol consumption, educational qualifications, occupation, ethnic group and income. 1.8 % of participants did not provide full details of their alcohol consumption, educational qualifications, occupation or ethnic group. These cases were excluded from the analyses. 20 % of participants did not provide income data, with larger proportions missing in lower SES groups. Multiple imputed complete datasets (m = 5) were generated using the other measures of SES and demographic variables and were used in analyses including equivalised income. After imputation, equivalised income quintiles were recalculated for each survey year.

Prevalence of each level of drinking was calculated and stratified by each SES indicator. Separate logistic regression models were used to test the association between each SES indicators and each drinking outcome variable. Odds ratios were adjusted for sex, age, ethnicity (aggregated using a classification designed to be consistent across Health Survey for England years [26]) and year of survey.

To test trends across SES levels for each drinking threshold, we coded each SES level numerically and used logistic regression to estimate an excess odds ratio for moving down one SES level. ‘Other’ categories in the education and occupation indicators were excluded. This provided a *p*-value for the hypothesis that the association between the ordered SES variable and the logit of exceeding the threshold was better described by a linear trend than no trend.

Health Survey for England survey weights, adjusting the sample according to non-response and the age, sex and regional structure of England’s population, were used in all analyses. R version 3.2.2 was used for data manipulation and analysis.

## Results

The analysis included 51,498 adults. 55 % were female and the mean age was 51 (sd 18). 90 % of the sample was from white ethnic groups (including white British and white Irish), 2 % was Indian, 2 % was Pakistani or Bangladeshi, 2 % was from black ethnic groups and 4 % was from mixed ethnic groups or ‘other’.

### Heavy weekly drinking

We first looked at heavy weekly drinking. 20.9 % of participants (95 % CI 20.4–21.5 %) reported exceeding the lowest threshold, 4.5 % (95 % CI 4.2–4.8 %) exceeded the second threshold, 1.5 % (95 % CI 1.3–1.6 %) exceeded the third threshold and 0.6 % (95 % CI 0.5–0.7 %) exceeded the most extreme threshold. Table 2 shows these prevalences stratified by SES indicators.

**Table 2:** Prevalence of heavy weekly drinking: percentage exceeding each threshold during a typical week, 2011–2013 (95 % CIs)

	Sample size	112 g+/168 g+	280 g+/400 g+	480 g+/640 g+	680 g+/880 g+
All	24409	20.9 (20.4–21.5)	4.5 (4.2–4.8)	1.5 (1.3–1.6)	0.6 (0.5–0.7)
Equivalised household income quintile					
> £49,000	4056	27.3 (25.9–28.8)	5.1 (4.4–5.8)	1.4 (1.0–1.8)	0.5 (0.3–0.7)
£29,000–£49,000	4201	24.2 (22.8–25.5)	4.4 (3.7–5.0)	1.4 (1.0–1.8)	0.5 (0.2–0.7)
£19,500–£29,900	3816	19.7 (18.5–21.0)	4.3 (3.7–4.9)	1.3 (0.9–1.7)	0.6 (0.3–0.8)
£12,800–£19,500	3822	17.3 (16.1–18.5)	4.3 (3.7–4.9)	1.5 (1.1–1.9)	0.6 (0.4–0.9)
< £12,800	3532	15.6 (14.4–16.9)	4.4 (3.7–5.2)	1.7 (1.2–2.2)	0.8 (0.5–1.1)
Highest qualification					
Degree	8855	23.2 (22.2–24.1)	4.2 (3.8–4.7)	1.3 (1.0–1.5)	0.5 (0.4–0.7)
A-level	3554	23.8 (22.4–25.4)	4.9 (4.2–5.7)	1.6 (1.2–2.1)	0.6 (0.4–1.0)
GCSE	5873	21.7 (20.6–22.9)	5.3 (4.7–5.9)	1.8 (1.5–2.2)	0.8 (0.6–1.1)
Other	386	16.3 (12.9–20.4)	2.3 (1.2–4.4)	0.3 (0.0–1.9)	0.0 (0.0–1.2)
None	5741	14.2 (13.2–15.2)	4.0 (3.5–4.6)	1.4 (1.1–1.8)	0.5 (0.4–0.8)
Occupation					
Higher managerial	4067	25.7 (24.2–27.1)	4.1 (3.5–4.7)	1.2 (0.9–1.6)	0.4 (0.2–0.7)
Lower managerial	5829	23.2 (22.0–24.4)	4.4 (3.8–5.0)	1.3 (1.0–1.7)	0.5 (0.3–0.7)
Intermediate	2720	20.2 (18.7–21.9)	5.0 (4.2–5.9)	1.5 (1.1–2.1)	0.7 (0.4–1.1)
Small employer/own account	2570	22.0 (20.4–23.8)	5.2 (4.4–6.2)	1.6 (1.1–2.2)	0.7 (0.4–1.1)
Lower supervisory/technical	2020	21.3 (19.5–23.3)	4.4 (3.5–5.4)	1.4 (1.0–2.1)	0.5 (0.3–1.0)
Semi-routine	3505	15.0 (13.8–16.3)	3.7 (3.1–4.5)	1.7 (1.2–2.2)	0.8 (0.5–1.3)
Routine	2960	17.5 (16.1–19.0)	5.1 (4.4–6.0)	1.9 (1.4–2.5)	0.8 (0.5–1.2)
Long-term unemployed	500	9.8 (7.3–13.1)	3.3 (2.0–5.4)	1.2 (0.5–2.7)	1.1 (0.4–2.6)
Other	238	23.4 (18.2–29.6)	7.2 (4.5–11.5)	1.3 (0.3–5.1)	0.7 (0.1–4.5)
Neighbourhood deprivation quintile					
1 (least deprived)	5128	23.8 (22.6–25.0)	4.9 (4.3–5.6)	1.2 (0.9–1.5)	0.5 (0.3–0.7)
2	5199	23.8 (22.6–25.0)	4.6 (4.0–5.2)	1.3 (1.0–1.7)	0.6 (0.4–0.9)
3	5101	20.9 (19.7–22.1)	4.2 (3.7–4.8)	1.4 (1.1–1.8)	0.4 (0.3–0.7)
4	4596	18.5 (17.4–19.7)	4.5 (3.9–5.2)	1.7 (1.3–2.2)	0.7 (0.4–1.0)
5 (most deprived)	4385	17.0 (15.8–18.2)	4.3 (3.7–5.0)	1.9 (1.5–2.4)	0.9 (0.7–1.3)

The income brackets for equivalised household income qunitile are for the 2013 survey

The sample size column is unweighted, while prevalence percentages have been calculated using post-stratification survey weights

Thresholds are grams of pure alcohol per week for women/men

**Fig. 1:**
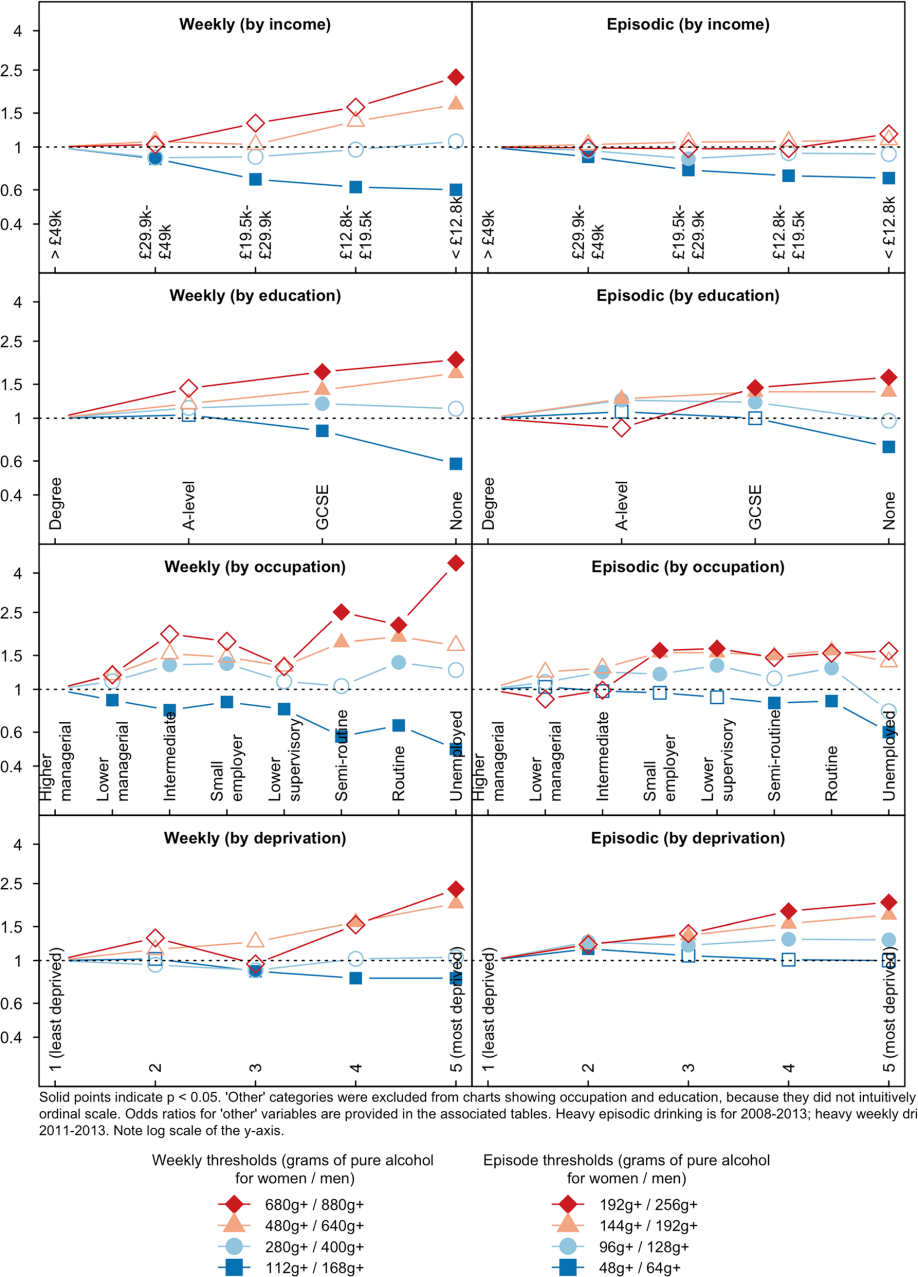
Odds ratio of exceeding drinking thresholds, compared to the highest status group. Adjusted for age, sex, ethnicity and year of survey

After adjusting for age, sex, ethnicity and year of survey, the patterns were similar for each SES indicator (see Tables 3 and 4). Higher SES groups were more likely to exceed the lowest threshold. The gradient reversed for the third and fourth thesholds, which lower SES participants were more likely to report exceeding. This is shown graphically in Fig. 1.

**Table 3:** Results of regression models: adjusted odds ratios of heavy weekly drinking, 2011–2013 (95 % CIs; *p*-values)

	112 g+/168 g+	280 g+/400 g+	480 g+/640 g+	680 g+/880 g+
Equivalised household income quintile				
> £49,000	1	1	1	1
£29,000–£49,000	0.87 (0.78–0.96; 0.007)	0.88 (0.72–1.07; 0.209)	1.07 (0.73–1.56; 0.748)	1.03 (0.52–2.04; 0.935)
£19,500–£29,900	0.68 (0.61–0.77;<0.001)	0.89 (0.73–1.10; 0.298)	1.03 (0.69–1.53; 0.898)	1.33 (0.71–2.49; 0.386)
£12,800–£19,500	0.62 (0.55–0.69;<0.001)	0.97 (0.79–1.19; 0.774)	1.36 (0.93–1.99; 0.115)	1.61 (0.85–3.03; 0.141)
< £12,800	0.60 (0.52–0.69;<0.001)	1.07 (0.84–1.35; 0.605)	1.66 (1.10–2.49; 0.015)	2.30 (1.28–4.13; 0.006)
Highest qualification				
Degree	1	1	1	1
A-level	1.04 (0.94–1.15; 0.457)	1.13 (0.92–1.38; 0.238)	1.19 (0.82–1.71; 0.366)	1.43 (0.82–2.49; 0.207)
GCSE	0.86 (0.79–0.93;<0.001)	1.19 (1.01–1.40; 0.039)	1.40 (1.04–1.89; 0.026)	1.74 (1.10–2.76; 0.017)
Other	0.76 (0.57–1.01; 0.059)	0.78 (0.39–1.54; 0.481)	0.50 (0.07–3.68; 0.504)	–
None	0.58 (0.52–0.64;<0.001)	1.12 (0.92–1.36; 0.272)	1.71 (1.20–2.44; 0.003)	2.01 (1.11–3.66; 0.022)
Occupation				
Higher managerial	1	1	1	1
Lower managerial	0.88 (0.79–0.98; 0.015)	1.10 (0.89–1.35; 0.402)	1.19 (0.79–1.78; 0.418)	1.19 (0.58–2.43; 0.645)
Intermediate	0.78 (0.69–0.89;<0.001)	1.34 (1.05–1.72; 0.017)	1.53 (0.98–2.39; 0.058)	1.93 (0.92–4.04; 0.079)
Small employer/own account	0.86 (0.76–0.97; 0.018)	1.36 (1.07–1.73; 0.012)	1.47 (0.93–2.32; 0.098)	1.77 (0.83–3.79; 0.141)
Lower supervisory/technical	0.79 (0.68–0.91;<0.001)	1.10 (0.83–1.45; 0.524)	1.32 (0.80–2.17; 0.279)	1.31 (0.55–3.15; 0.553)
Semi-routine	0.57 (0.50–0.65;<0.001)	1.04 (0.81–1.34; 0.762)	1.75 (1.14–2.68; 0.010)	2.51 (1.22–5.18; 0.012)
Routine	0.65 (0.57–0.74;<0.001)	1.38 (1.09–1.75; 0.008)	1.87 (1.22–2.87; 0.004)	2.15 (1.06–4.36; 0.035)
Long-term unemployed	0.49 (0.35–0.69;<0.001)	1.26 (0.73–2.19; 0.409)	1.69 (0.68–4.19; 0.258)	4.51 (1.52–13.43; 0.007)
Other	1.17 (0.83–1.64; 0.375)	2.30 (1.35–3.94; 0.002)	1.39 (0.34–5.71; 0.657)	2.08 (0.28–15.60; 0.488)
Neighbourhood deprivation quintile				
1 (least deprived)	1	1	1	1
2	1.02 (0.93–1.13; 0.664)	0.95 (0.78–1.15; 0.597)	1.14 (0.78–1.67; 0.504)	1.31 (0.70–2.42; 0.404)
3	0.88 (0.80–0.98; 0.016)	0.89 (0.73–1.08; 0.226)	1.25 (0.86–1.81; 0.246)	0.96 (0.51–1.81; 0.916)
4	0.81 (0.73–0.90;<0.001)	1.02 (0.83–1.25; 0.858)	1.58 (1.08–2.31; 0.017)	1.53 (0.83–2.80; 0.170)
5 (most deprived)	0.81 (0.72–0.91;<0.001)	1.04 (0.84–1.28; 0.742)	1.97 (1.36–2.86;<0.001)	2.34 (1.34–4.11; 0.003)

Adjusted for age, sex, ethnicity and year of survey

The income brackets for equivalised household income qunitile are for the 2013 survey

Thresholds are grams of pure alcohol per week for women/men

**Table 4:** Tests for linear trend in log odds of exceeding heavy weekly drinking thresholds: adjusted excess odds ratios of moving down one SES level, 2011–2013 (95 % CI; *p*-value)

	112 g+/168 g+	280 g+/400 g+	480 g+/640 g+	680 g+/880 g+
Income	0.87 (0.84–0.90;<0.001)	1.02 (0.96–1.08; 0.490)	1.14 (1.03–1.25; 0.010)	1.24 (1.08–1.43; 0.003)
Education	0.88 (0.86–0.90;<0.001)	1.03 (0.99–1.08; 0.163)	1.15 (1.05–1.25; 0.002)	1.19 (1.04–1.37; 0.011)
Occupation	0.92 (0.91–0.94;<0.001)	1.03 (1.00–1.06; 0.071)	1.09 (1.04–1.15; 0.001)	1.15 (1.06–1.25; 0.001)
Deprivation	0.94 (0.91–0.96;<0.001)	1.01 (0.97–1.06; 0.608)	1.19 (1.09–1.29;<0.001)	1.22 (1.06–1.40; 0.005)

Adjusted for age, sex, ethnicity and year of suvey

‘Other’ categories were excluded from education and occupation

Note that SES indicators have different numbers of levels. For example, ‘deprivation’ is based on quintiles, so an excess odds ratio of 1.22 represents an estimated odds ratio of 2.22 comparing bottom and top quintiles. Occupation has eight levels, so an excess odds ratio of 1.15 represents an estimated odds ratio of 2.66 between ‘unemployed’ and ‘higher managerial’

Thresholds are grams of pure alcohol per week for women/men

There were strong associations between the lowest levels of SES and exceeding the highest drinking threshold. For example, long-term unemployed participants had 4.51 (95 % CI 1.52–13.43; *p* = 0.007) times the odds of exceeding this threshold when compared to those with ‘higher managerial’ occupations. Participants living in the most deprived neighbourhoods had 2.34 (95 % CI 1.34–4.11; *p* = 0.003) times the odds of exceeding this threshold when compared to those living in the least deprived neighbourhoods.

### Heavy episodic drinking

We then looked at heavy episodic (‘binge’) drinking. 18.3 % of participants (95 % CI 17.9–18.6 %) reported exceeding the lowest threshold, 6.9 % (95 % CI 6.6–7.1 %) exceeded the second threshold, 2.3 % (95 % CI 2.1–2.4 %) exceeded the third threshold and 0.8 % (95 % CI 0.7–0.9 %) exceeded the most extreme threshold. Table 5 shows these prevalences stratified by SES indicators.

**Table 5:** Prevalence of heavy episodic drinking: percentage exceeding each threshold on the heaviest day in the past week, 2008–2013 (95 % CIs)

	Sample size	48 g+/64 g+	96 g+/128 g+	144 g+/192 g+	192 g+/256 g+
All	51498	18.3 (17.9–18.6)	6.9 (6.6–7.1)	2.3 (2.1–2.4)	0.8 (0.7–0.9)
Equivalised household income quintile					
> £49,000	8879	23.6 (22.5–24.6)	8.0 (7.4–8.7)	2.3 (2.0–2.7)	0.8 (0.6–1.0)
£29,000–£49,000	8750	21.1 (20.2–22.0)	7.6 (7.0–8.2)	2.4 (2.0–2.8)	0.8 (0.6–1.1)
£19,500–£29,900	8080	17.2 (16.3–18.0)	6.4 (5.8–6.9)	2.2 (1.9–2.6)	0.7 (0.5–0.9)
£12,800–£19,500	8140	14.7 (13.9–15.5)	6.1 (5.5–6.6)	2.1 (1.7–2.4)	0.7 (0.5–0.9)
< £12,800	7268	14.3 (13.5–15.1)	6.1 (5.6–6.7)	2.2 (1.9–2.6)	0.8 (0.6–1.0)
Highest qualification					
Degree	17479	20.0 (19.4–20.7)	6.7 (6.3–7.1)	2.0 (1.8–2.3)	0.6 (0.5–0.7)
A-level	7413	24.4 (23.3–25.5)	10.8 (10.0–11.6)	3.6 (3.2–4.1)	0.8 (0.7–1.1)
GCSE	13011	20.3 (19.5–21.0)	8.1 (7.6–8.6)	2.8 (2.5–3.1)	0.9 (0.8–1.1)
Other	870	6.2 (4.7–8.1)	1.7 (1.0–2.9)	0.3 (0.1–1.2)	0.0 (0.0–0.5)
None	12725	9.6 (9.1–10.2)	3.4 (3.0–3.7)	1.3 (1.1–1.5)	0.4 (0.3–0.5)
Occupation					
Higher managerial	8213	20.0 (19.1–21.0)	6.3 (5.7–6.9)	1.8 (1.4–2.1)	0.6 (0.4–0.9)
Lower managerial	12600	20.2 (19.5–21.0)	6.8 (6.3–7.3)	2.1 (1.9–2.5)	0.6 (0.4–0.7)
Intermediate	5197	17.8 (16.6–19.0)	7.0 (6.2–7.8)	2.1 (1.7–2.6)	0.6 (0.4–0.9)
Small employer/own account	5535	17.8 (16.7–18.9)	6.6 (5.9–7.3)	2.4 (1.9–2.8)	0.9 (0.6–1.2)
Lower supervisory/technical	4783	18.0 (16.8–19.2)	7.9 (7.1–8.8)	2.7 (2.2–3.2)	1.1 (0.8–1.5)
Semi-routine	7233	15.4 (14.5–16.4)	6.4 (5.8–7.1)	2.4 (2.0–2.9)	0.9 (0.7–1.2)
Routine	6491	16.5 (15.5–17.5)	7.3 (6.6–8.0)	2.6 (2.2–3.1)	0.9 (0.7–1.2)
Long-term unemployed	1011	9.3 (7.6–11.5)	3.6 (2.6–5.0)	1.9 (1.2–2.9)	0.8 (0.4–1.5)
Other	435	29.4 (24.9–34.3)	16.5 (13.1–20.7)	4.6 (2.9–7.4)	1.0 (0.4–2.7)
Neighbourhood deprivation quintile					
1 (least deprived)	11106	17.7 (17.0–18.5)	5.7 (5.2–6.2)	1.6 (1.4–1.9)	0.5 (0.4–0.7)
2	10682	19.6 (18.8–20.5)	7.0 (6.5–7.6)	2.0 (1.7–2.3)	0.6 (0.5–0.8)
3	10538	18.9 (18.1–19.7)	7.0 (6.5–7.6)	2.3 (2.0–2.7)	0.7 (0.6–1.0)
4	9864	18.1 (17.2–18.9)	7.6 (7.0–8.2)	2.7 (2.4–3.1)	1.0 (0.8–1.2)
5 (most deprived)	9308	16.8 (16.0–17.6)	7.2 (6.6–7.8)	2.9 (2.5–3.3)	1.0 (0.8–1.3)

The income brackets for equivalised household income qunitile are for the 2013 survey

The sample size column is unweighted, while prevalence percentages have been calculated using post-stratification survey weights

Thresholds are grams of pure alcohol per week for women/men

Adjusted analyses based on education and occupation showed similar patterns to those observed for heavy weekly drinking (see Table 6 and 7). Those with higher-level qualifications and higher status occupations were more likely to exceed the lowest threshold, while participants with lower-level qualifications and lower status occupations were more likely to exceed the more extreme thresholds.

**Table 6:** Results of regression models: adjusted odds ratios of heavy episodic drinking, 2008–2013 (95 % CIs; *p*-values)

	48 g+/64 g+	96 g+/128 g+	144 g+/192 g+	192 g+/256 g+
Equivalised household income quintile				
> £49,000	1	1	1	1
£29,000–£49,000	0.89 (0.82–0.96; 0.003)	0.96 (0.85–1.09; 0.513)	1.03 (0.83–1.29; 0.787)	0.99 (0.67–1.46; 0.968)
£19,500–£29,900	0.76 (0.70–0.82;<0.001)	0.87 (0.77–0.98; 0.027)	1.06 (0.83–1.34; 0.668)	0.98 (0.67–1.43; 0.923)
£12,800–£19,500	0.71 (0.64–0.77;<0.001)	0.93 (0.82–1.05; 0.250)	1.07 (0.84–1.37; 0.588)	0.98 (0.63–1.51; 0.925)
< £12,800	0.69 (0.63–0.76;<0.001)	0.92 (0.80–1.06; 0.240)	1.09 (0.86–1.38; 0.481)	1.17 (0.78–1.76; 0.460)
Highest qualification				
Degree	1	1	1	1
A-level	1.08 (1.00–1.16; 0.053)	1.24 (1.11–1.39;<0.001)	1.26 (1.05–1.52; 0.015)	0.89 (0.63–1.25; 0.513)
GCSE	1.00 (0.93–1.06; 0.915)	1.21 (1.10–1.34;<0.001)	1.37 (1.15–1.62;<0.001)	1.44 (1.09–1.91; 0.010)
Other	0.73 (0.54–0.98; 0.035)	1.01 (0.58–1.76; 0.966)	0.79 (0.19–3.28; 0.758)	–
None	0.71 (0.65–0.77;<0.001)	0.97 (0.85–1.11; 0.715)	1.37 (1.09–1.72; 0.007)	1.63 (1.09–2.43; 0.016)
Occupation				
Higher managerial	1	1	1	1
Lower managerial	1.03 (0.95–1.12; 0.464)	1.09 (0.96–1.24; 0.179)	1.23 (0.97–1.56; 0.093)	0.89 (0.58–1.37; 0.599)
Intermediate	0.98 (0.88–1.08; 0.672)	1.23 (1.04–1.46; 0.014)	1.29 (0.96–1.73; 0.091)	0.99 (0.59–1.68; 0.979)
Small employer/own account	0.96 (0.87–1.06; 0.431)	1.20 (1.03–1.41; 0.022)	1.55 (1.17–2.05; 0.002)	1.59 (1.00–2.54; 0.049)
Lower supervisory/technical	0.91 (0.82–1.02; 0.095)	1.33 (1.13–1.57;<0.001)	1.55 (1.16–2.06; 0.003)	1.63 (1.01–2.63; 0.044)
Semi-routine	0.85 (0.77–0.93;<0.001)	1.14 (0.98–1.33; 0.090)	1.50 (1.15–1.95; 0.003)	1.46 (0.93–2.30; 0.098)
Routine	0.87 (0.79–0.96; 0.006)	1.29 (1.11–1.50;<0.001)	1.59 (1.22–2.07;<0.001)	1.54 (0.98–2.42; 0.058)
Long-term unemployed	0.60 (0.47–0.77;<0.001)	0.77 (0.54–1.09; 0.142)	1.39 (0.84–2.30; 0.202)	1.58 (0.70–3.58; 0.277)
Other	1.44 (1.13–1.85; 0.004)	1.91 (1.40–2.60;<0.001)	1.42 (0.82–2.47; 0.210)	0.74 (0.25–2.14; 0.584)
Neighbourhood deprivation quintile				
1 (least deprived)	1	1	1	1
2	1.15 (1.07–1.25;<0.001)	1.25 (1.10–1.42;<0.001)	1.22 (0.96–1.54; 0.098)	1.21 (0.80–1.84; 0.374)
3	1.06 (0.98–1.15; 0.128)	1.20 (1.05–1.36; 0.005)	1.35 (1.08–1.69; 0.009)	1.38 (0.93–2.04; 0.108)
4	1.01 (0.93–1.10; 0.767)	1.29 (1.14–1.47;<0.001)	1.55 (1.24–1.93;<0.001)	1.80 (1.23–2.63; 0.002)
5 (most deprived)	1.00 (0.92–1.09; 0.966)	1.28 (1.12–1.45;<0.001)	1.72 (1.38–2.15;<0.001)	2.00 (1.36–2.93;<0.001)

Adjusted for age, sex, ethnicity and year of survey

The income brackets for equivalised household income qunitile are for the 2013 survey

Thresholds are grams of pure alcohol per week for women/men

**Table 7:** Tests for linear trend in log odds of exceeding heavy episodic drinking thresholds: adjusted excess odds ratios of moving down one SES level, 2008–2013 (95 % CI; *p*-value)

	48 g+/64 g+	96 g+/128 g+	144 g+/192 g+	192 g+/256 g+
Income	0.91 (0.89–0.93;<0.001)	0.98 (0.95–1.01; 0.187)	1.03 (0.98–1.09; 0.242)	1.03 (0.94–1.13; 0.560)
Education	0.93 (0.91–0.95;<0.001)	1.01 (0.98–1.04; 0.425)	1.09 (1.04–1.15;<0.001)	1.16 (1.05–1.27; 0.002)
Occupation	0.97 (0.95–0.98;<0.001)	1.03 (1.01–1.05; 0.004)	1.07 (1.03–1.10;<0.001)	1.10 (1.05–1.16;<0.001)
Deprivation	0.99 (0.97–1.01; 0.192)	1.05 (1.02–1.08;<0.001)	1.14 (1.09–1.20;<0.001)	1.19 (1.10–1.30;<0.001)

Adjusted for age, sex, ethnicity and year of survey

‘Other’ categories were excluded from education and occupation

Note that SES indicators have different numbers of levels. For example, ‘deprivation’ is based on quintiles, so an excess odds ratio of 1.19 represents an estimated odds ratio of 2.01 comparing bottom and top quintiles. Occupation has eight levels, so an excess odds ratio of 1.10 represents an estimated odds ratio of 1.95 between ‘unemployed’ and ‘higher managerial’

Thresholds are grams of pure alcohol in one day for women/men

The pattern was slightly different for income and neighbourhood deprivation. Participants with higher incomes were more likely to exceed the lowest threshold, and while the gradient reduced at higher thresholds, it did not reverse and there was no gradient for the second, third and highest thresholds.

Neighbourhood deprivation was the only measure where no gradient was observed for the lowest threshold (i.e., each quintile had similar odds of exceeding this threshold). The gradients then steepened, with participants in more deprived neighbourhoods more likely to exceed the second, third and most extreme thresholds. Those in the most deprived neighbourhoods had 2.00 (95 % CI 1.36–2.93) times the odds of exceeding the highest threshold when compared to those in the least deprived. This is shown graphically in Fig. 1.

## Discussion

This study examined the social gradients of a range of definitions of heavy drinking, both in terms of weekly and episodic high-intensity consumption. High SES groups were more likely to report exceeding the lowest thresholds of regular heavy or high intensity drinking, while lower SES groups were more likely to exceed the more extreme thresholds. These patterns were consistent across indicators of SES based on income, education, occupation and neighbourhood deprivation. This ‘reversal’ in gradient reflects diversity in drinking levels in the low SES groups, which include more abstainers and light drinkers as well as more extreme drinkers.

Alcohol-related harm is likely to be severe in the group reporting the most extreme drinking levels. One reason for this is the ‘J-shaped curve’ in alcohol-related harm, which is the theory that light and moderate drinking is cardioprotective and reduces all-cause mortality [27]. Although the beneficial effects have been questioned, [28] the theory suggests that alcohol-related harm is concentrated in more extreme drinkers. This study shows that these drinkers are disproportionately of low SES. Furthermore, other risk factors, such as smoking, poor diet, overweight and physical inactivity have been shown to cluster in low SES drinkers, [17] which could act multiplicatively with alcohol. These factors suggest greater vulnerability to alcohol-related harm in low SES groups and may partly explain why they experience higher alcohol-related mortality and morbidity, and hence the alcohol harm paradox.

Many other studies have observed that individuals in lower SES groups are less likely to report exceeding the lowest drinking thresholds used in this study [4, 7–11]. However, few studies have considered more extreme thresholds of alcohol use and there is little evidence to compare our results against. Consistent with our findings, a study in the Netherlands observed that both abstinence and ‘excessive drinking’ (defined similarly to our second threshold of episodic drinking) were most common in participants with the lowest level of education [15]. Studies in Wales [29] and England [17] found that ‘binge drinking’ was associated with living in a deprived neighbourhood, which reflects the lack of gradient across neighbourhood deprivation quintiles for lowest threshold of heavy episodic drinking in our study. Long-term household unemployment is a strong marker of SES and was very strongly associated with extreme weekly drinking in our study. Likewise, other research has observed a strong relationship between unemployment and alcohol-related mortality [30].

Our findings are based on a sample from England, and are likely to be relevant in industrialised countries where lower SES groups have higher rates of alcohol-related harm that cannot be explained by reported differences in consumption. Most indicators of low SES in these countries are associated with low average rates of drinking, driven at least in part by higher rates of abstention.

The study was based on large, nationally representative surveys that allow reasonably precise estimation of extreme drinking. However, it had limitations regarding its cross-sectional design, potential selection bias and the self-report alcohol measure.

First, the study is cross-sectional and so does not provide evidence of a causal relationship between SES and alcohol consumption. By focusing on drinking patterns, it does not provide explicit insight into the multifaceted relationship between extreme drinking and health. The study examines current drinking rather than drinking history, and therefore does not distinguish, for example, between lifetime abstainers and quitters.

Second, the Health Survey for England may have selection bias as a result of exclusion from the sample frame or non-response. Excluded groups include inpatients, homeless people, people living in hostels and students in university halls. Other studies have shown that surveys of private households disproportionately under-represent dependent drinkers [12, 31]. This is likely to mean that the data under-represent lower status heavy drinkers and that the reversal in gradient we report here is actually underestimated. The extent of non-response bias is difficult to estimate and may not have been fully corrected by the use of survey weights. Studies of non-response and alcohol have mixed results. They most commonly show that non-responders are slightly heavier drinkers, but this is not strongly associated with SES [32]. There appears to be a low risk of non-response bias. It is most likely to have caused underestimation of the odds of lower status groups exceeding each threshold when compared with higher status groups (again suggesting our estimates are conservative).

Third, alcohol consumption is based on self-report, which commonly underestimates drinking. The mean weekly consumption reported in this study’s sample from 2011 to 2013 was 87 g. This is similar to the 92 g reported in the 2011 General Lifestyle Survey, [33] which is also based on self-report. Both estimates are much lower than the 149 g per week calculated from UK tax revenue in 2011/12 [34]. A study that compared recall with a seven-day drinking diary found that underreporting was greater for people who drank more, but did not differ by SES [33]. Similar findings were produced by a study comparing recall with detailed telephone interviews including special occasions (such as Christmas) [35]. Prevalence of all drinking levels in this study is likely to be underestimated, with greater underestimation of the heaviest levels. However, inaccuracies in self-report are unlikely to invalidate the social gradients observed in this study.

## Conclusions

This study showed that individuals in low SES groups have divergent drinking patterns and are more likely to report extreme drinking. This is an important finding for public health policy. Although the group that reported extreme drinking is small (for example, 2.5 % of participants in the most deprived neighbourhoods reported exceeding the third threshold of heavy weekly drinking and 1.0 % the fourth), it may have a large impact on the social distribution of disease burden and health service use. This group is likely to have high rates of unemployment, mental health problems, low resilience and other adverse life circumstances.

There is limited understanding of how interventions to reduce alcohol-related harm affect this group. For example, modelling suggests that minimum unit pricing would be of both financial and health benefit to ‘harmful drinkers’ in the lowest income quintile, [36] but harmful drinkers are defined as those exceeding only the second threshold of weekly drinking in our study. The small group of more extreme drinkers, who are likely to contribute disproportionately to alcohol-related health inequalities, could behave differently. Similarly, the value of screening and behavioural-based interventions, such as the NHS Health Checks programme in the UK, [37] is unclear for extreme drinkers in low SES groups. Further research is important given the group’s likely vulnerability, high rates of alcohol-related harm and contribution to healthcare costs.

The divergent drinking patterns in low SES groups highlights the need for more disaggregated health promotion. Long-term unemployed men reporting more than 880 g of alcohol consumption per week (equivalent to daily drinking of over half a 70 cl bottle of spirits or eight 500 ml cans of beer), for example, are likely to respond to interventions differently to less extreme drinkers and groups with greater social capital. Policies to address alcohol-related health inequalities may require specific focus on extreme drinkers who are living in poverty.

## Abbreviations

CI, Confidence Interval; OR, Odds Ratio, SES Socioeconomic Status.
